# COVID-19 Induced Acute Pancreatitis: A Case Report and Literature Review

**DOI:** 10.7759/cureus.9169

**Published:** 2020-07-13

**Authors:** Saurabh Kataria, Aftab Sharif, Asad Ur Rehman, Zahoor Ahmed, Abdul Hanan

**Affiliations:** 1 Neurology, University of Missouri, Columbia, USA; 2 General Medicine, King Edward Medical University, Mayo Hospital, Lahore, PAK; 3 Internal Medicine, Pakistan Kidney and Liver Research Institute, Lahore, PAK; 4 Internal Medicine, King Edward Medical University, Mayo Hospital, Lahore, PAK; 5 Infectious Disease, Wayne State University, Detroit, USA

**Keywords:** covid-19, coronavirus, pancreatitis

## Abstract

A 49-year-old female with no history of past medical illness presented to the emergency department with complaints of fever, dry cough, and shortness of breath. Initial evaluation revealed a temperature of 101°F, and on auscultation, the patient had scattered wheezing and rales in left lung fields. CT of the chest revealed pneumonic patches in the upper and lower segment of the left lung. Her COVID-19 testing came positive. On the second day of hospital admission, the patient experienced nausea, vomiting, and severe epigastric pain radiating to back. Laboratory analysis revealed a marked elevation of lipase and amylase. CT of the abdomen showed an edematous pancreas with diffuse enlargement. She was diagnosed with acute pancreatitis due to COVID-19 after carefully ruling out other causes. She was managed symptomatically, and improvement in her clinical condition was observed and was discharged with outpatient follow-up. Although acute pancreatitis is rare in patients with COVID-19, it should be considered as a differential diagnosis in patients with severe epigastric pain and respiratory symptoms.

## Introduction

Fever, cough, dyspnea, sore throat, headache, and myalgia are the characteristic symptoms of COVID-19 [1]. Although COVID-19 has been highlighted principally to affect the respiratory system, gastrointestinal (GI) involvement has also been underlined in the published literature. The GI manifestations of the COVID-19 infection include anorexia, nausea, vomiting, abdominal pain, and diarrhea. However, pancreatic involvement in patients with COVID-19 is rarely reported. Pancreatic symptomology usually corresponds to asymptomatic abnormal pancreatic enzyme elevation and acute pancreatitis. Here, we present a rare case of acute pancreatitis in a patient with COVID-19.

## Case presentation

**Figure 1:**
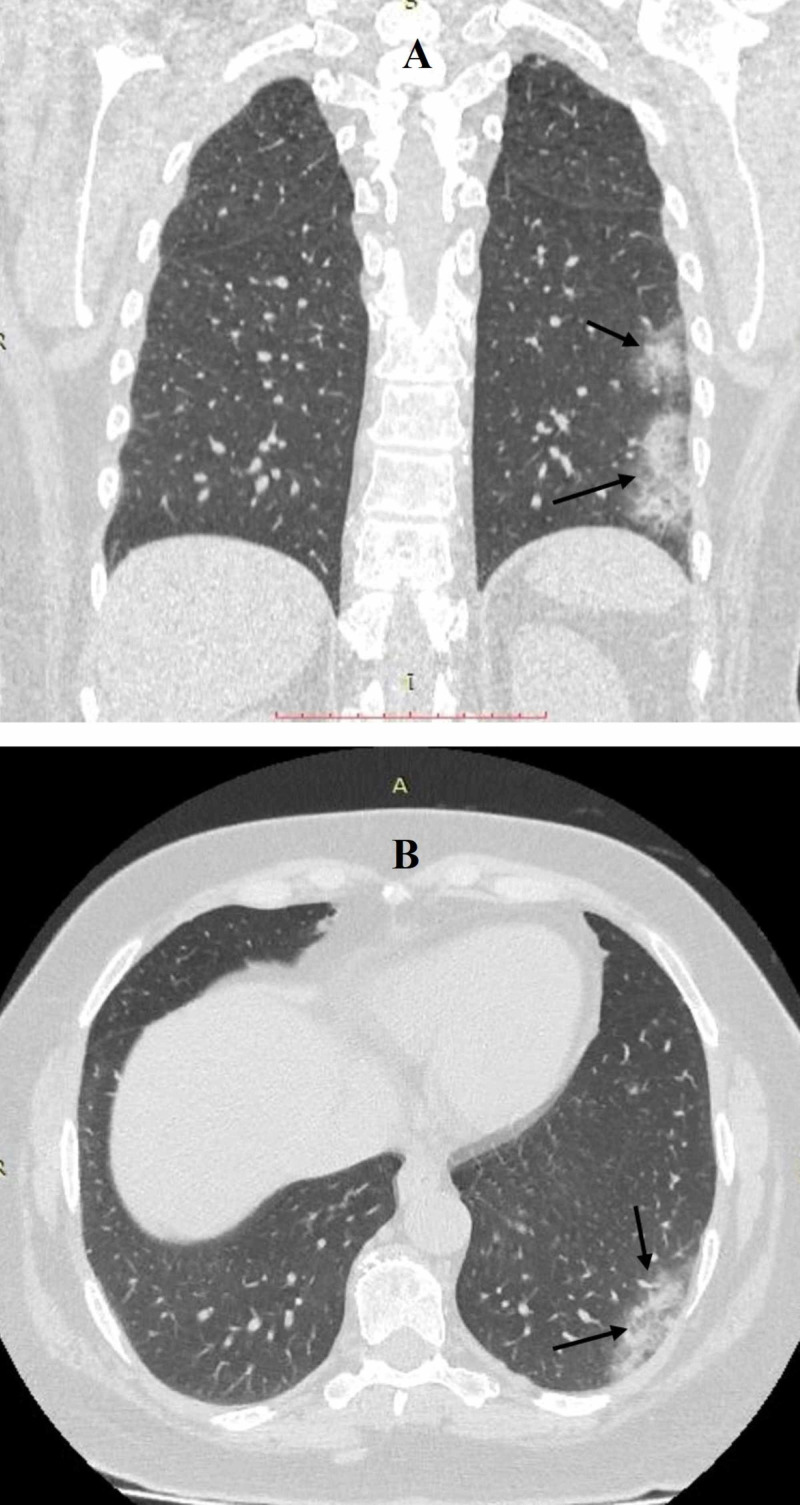
Coronal (A) and axial (B) sections of CT chest showing infiltrates in the apico-posterior and posterior basal segments of the left lung.

A 49-year-old female with no past medical history presented to the emergency department with complaints of fever, dry cough, lethargy, and shortness of breath for the last four days. The initial evaluation revealed a temperature of 101°F, a blood pressure of 110/70 mmHg, a heart rate of 108 beats per minute, a respiratory rate of 20 per minute, and an oxygen saturation of 89% on room air. Physical exam revealed a severe dry cough, and on auscultation, there were scattered wheezing and rales in left lung fields. The rest of the physical exam was nonsignificant. A chest X-ray revealed small pneumonic patches in the upper and lower segments of the left lung. The patient was tested for COVID-19. She was admitted with a possible diagnosis of acute pneumonia and was isolated for suspected COVID-19. CT of the chest was performed, which revealed multifocal infiltrates involving the posterior basal segment of the left lower lobe and an apico-posterior segment of the left upper lobe (Figure 1). On initial laboratory analysis, the patient’s white blood cell count, platelet count, and hematocrit were within normal ranges except for elevated for C-reactive protein (25.1 mg/L). She was started on intravenous ceftriaxone 1 g twice a day, and intravenous azithromycin 500 mg daily with high-flow oxygen supplementation due to hypoxic respiratory failure, and on the next day, her COVID-19 testing was positive.

On second day of admission, the patient developed severe epigastric pain radiating to back, followed by nausea and one episode of vomiting containing food particles. Abdominal examination revealed epigastric tenderness. Laboratory analysis was unremarkable except for the marked elevation of lipase and amylase (Table 1). Her low-density lipoprotein (LDL) was 61 mg/dL, high-density lipoprotein (HDL) 49 mg/dL, serum cholesterol 191 mg/dL, and serum triglyceride 153 mg/dL. Abdominal ultrasonography revealed normal-sized liver and biliary ducts with no evidence of gallstone. She had no history of alcoholism, smoking, and drug abuse.

**Table 1:** Comprehensive metabolic panel on the second day of admission.

Parameter	Lab value	Normal range
Lipase (IU/L)	1,541	0–160
Amylase (IU/L)	501	30–110
Alkaline phosphatase (mg/dL)	80	36–92
Aspartate aminotransferase (IU/L)	40	8–48
Alanine aminotransferase (IU/L)	52	7–55
Prothrombin time (second)	12.1	11–13.5
Partial thromboplastin time (second)	32	30–40
Total bilirubin (mg/dL)	2.1	0.3–1.2
C-reactive protein (mg/L)	25.1	<10
Sodium (mmol/L)	139	136–145
Potassium (mmol/L)	3.8	3.5–5.0
Chloride (mmol/L)	99	98–106
Urea nitrogen (mg/dL)	13	8–20
Creatinine (mg/dL)	1.5	07–1.2
Blood glucose (mg/dL)	96	70–100 (Fasting)
Calcium (mg/dL)	8.6	9.0–10.5

**Figure 2:**
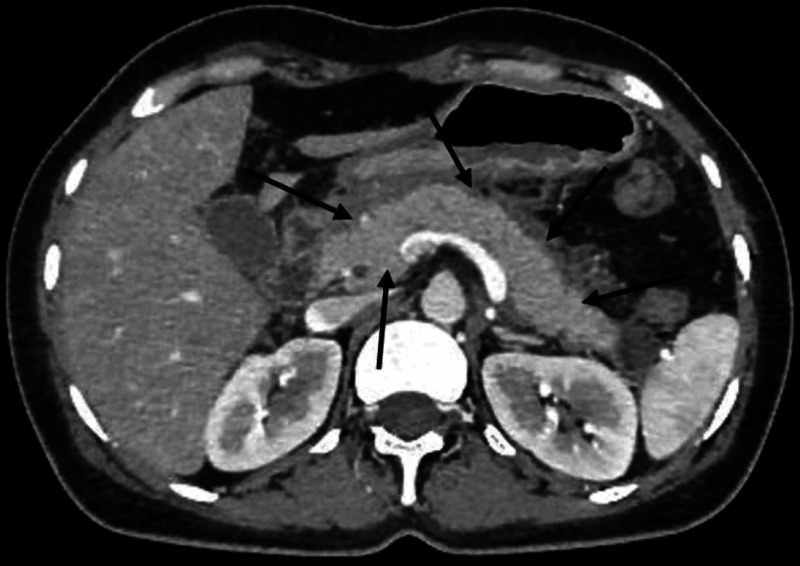
Transverse section of CT abdomen showing diffuse enlargement of pancreas with ill-defined borders and surrounding peripancreatic fluid.

She was diagnosed with acute pancreatitis due to COVID-19, as she had no other risk factors for acute pancreatitis. She was admitted to the intensive care unit and was managed symptomatically with intravenous fluids and analgesia. Her CT abdomen revealed an edematous pancreas with diffuse enlargement and ill-defined border (Figure 2). Supportive treatment continued, and improvement in the clinical condition of the patient was observed. Her temperature subsided gradually. Oxygen requirement declined over the next seven days with gradual improvement in her pulmonary and GI symptoms and was discharged with outpatient follow-up.

## Discussion

COVID-19 has been widely studied as a lung pathogen. However, GI association with COVID-19 has also been reported in recent studies. Studies have shown that up to 50% of COVID-19 patients develop GI symptoms of nausea, vomiting, diarrhea, and abdominal pain [2]. In a recent study, 11.4% of the patients exhibited at least one of the GI symptoms, involving nausea, vomiting, or diarrhea [3]. However, pancreatic involvement is rare. Pancreatic symptomology usually corresponds to asymptomatic abnormal pancreatic enzyme elevation or acute pancreatitis in COVID-19. A recent study by Wang et al. reported that out of 52 patients with COVID-19, eight patients experienced pancreatic injury (abnormal elevation in lipase or amylase) [4]. However, these patients did not experience the clinical symptoms of acute pancreatitis. Acute pancreatitis as a presenting sign has been reported in very few case reports in the literature [5].

The mechanism of pancreatic injury in COVID-19 is due to the expression of angiotensin-converting enzyme 2 (ACE2) receptors in pancreatic cells. Glycosylated-spike (S) protein is one of the structural proteins encoded by the coronavirus genome, and it is a prime inducer of the host immune response. This protein binds to ACE2 receptor protein located on the host cell surface membrane and mediates the host cell invasion [6-8]. ACE2 does not only express in the lung alveolar type-2 (AT-2) cells but also manifests in the esophagus, small intestine, large intestine, and pancreatic islets cells [9]. High expression of ACE2 receptors in the pancreatic islet cells can cause cell damage due to COVID-19, resulting in acute pancreatitis. Direct cytopathic effects of COVID-19 or immune-mediated and indirect systemic inflammatory response could be the mechanism of pancreatic injury [10,11]. Antipyretics, which most of the patients take before admission, could also cause drug-induced pancreatitis. However, our patient did not use any medication before presenting to the hospital.

Diagnosis of acute pancreatitis lies in the clinical presentation of the patient, lab values, and the use of imaging modalities. Two of the following three criteria must be met to diagnose acute pancreatitis: (1) upper abdominal pain consistent with the disease activity (acute onset, epigastric, and usually radiating to back); (2) serum Lipase or amylase level > 3x the upper limit of normal; (3) distinctive acute pancreatitis findings on imaging modalities (such as ultrasonography, abdominal CT, or MRI) [12,13]. Acute pancreatitis is managed symptomatically, supportive care with fluids and analgesia, and nutritional support with enteral nutrition, if the patient cannot tolerate an oral diet. The use of prophylactic antibiotics is usually prohibited in acute pancreatitis [14].

Our patient was previously healthy, with no history of alcohol or drug abuse. Similarly, abdominal ultrasonography revealed no evidence of gallstone. Furthermore, hypertriglyceridemia as a possible trigger was not considered due to the serum triglycerides level of 153 mg/dL shown on the lipid panel. She did not take any antipyretics, which might be the cause of acute pancreatitis. Her improvement with conservative management confirmed acute pancreatitis attributable to COVID-19.

## Conclusions

COVID-19 infection is a pressing concern due to the involvement of the GI system, especially the pancreas. COVID-19 induced acute pancreatitis is a rare cause, and other common causes of acute pancreatitis must be ruled out thoroughly. Severe acute respiratory syndrome coronavirus 2 (SARS-CoV-2) has complex presentations because of multisystem involvement and can be lethal if not identified and addressed appropriately. Therefore, clinicians should have sound knowledge and pay close attention to SARS-CoV-2 infection and related pancreatic complications.
